# Large‐Scale Growth of Self‐Poled Ferroelectric Rashba Semiconductor *α*‐GeTe(111) Thin Films: A Crucial Step Towards Future CMOS‐Compatible Ferroelectric Spintronic Devices

**DOI:** 10.1002/advs.75711

**Published:** 2026-05-22

**Authors:** Jules Lagrave, Nicolas Bernier, Thomas Jalabert, Yoann Brûlé, Damien Térébénec, Pierre Meilleur, Hervé Roussel, Jean‐Baptiste Dory, Oussama J. Mouawad, Thibault Chommaux, Cristian Mocuta, Dominique Thiaudière, Laurent Vila, Françoise Hippert, Pierre Noé

**Affiliations:** ^1^ Université Grenoble Alpes, CEA, LETI Grenoble France; ^2^ Université Grenoble Alpes, CNRS, Grenoble INP, LMGP Grenoble France; ^3^ Synchrotron SOLEIL, l'Orme des Merisiers Gif‐sur‐Yvette France; ^4^ Université Grenoble Alpes, CEA, CNRS, Grenoble INP, SPINTEC Grenoble France

**Keywords:** chalcogenides, ferroelectrics, GeTe, spintronics, sputtering, van der Waals epitaxy

## Abstract

Chalcogenide ferroelectric Rashba semiconductors (FERSCs) offer a promising route to ultralow‐power spin–orbit devices, yet their integration into microelectronics has been hindered by the lack of high‐quality films grown using industry‐compatible methods. Here, we demonstrate CMOS‐compatible synthesis of rhombohedral *α*‐GeTe(111) thin films on 200/300 mm Si wafers using an industrial deposition process based on van der Waals epitaxy, applied to this material class for the first time. Scanning Transmission Electron microscopy and advanced simulations of anomalous X‐ray diffraction measurements at synchrotron reveal that films are intrinsically self‐poled, exhibiting a robust upward out‐of‐plane ferroelectric polarization. The growth strategy is universal, enabling high‐quality *α*‐GeTe(111) on metals and insulators without compromising structural or ferroelectric performance. Piezoresponse force microscopy confirms reversible 180° ferroelectric switching with performance comparable to molecular‐beam‐epitaxy benchmarks. This substrate‐independent growth strategy represents a decisive step toward the future large‐scale integration of FERSCs into functional spin‐orbit architectures, with the hope of bridging the gap between Rashba's fundamental physics and microelectronic implementation.

## Graphical abstract

Ferroelectric Rashba semiconductors promise ultralow‐power devices but lack industry‐quality films. This work demonstrates CMOS‐compatible fabrication of high‐quality *α*‐GeTe(111) films via magnetron sputtering, enabled by a 5 nm Sb_2_Te_3_ seed layer. Structural and ferroelectric analyses show robust, switchable polarization comparable to MBE films, paving the way for scalable spin‐orbit memory and logic technologies.

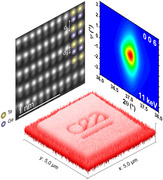

## 1 Introduction

The increasing energy demands of modern electronics call for new concepts that offer non‐volatility, low power consumption, and high‐speed operation while remaining compatible with conventional complementary metal‐oxide‐semiconductor (CMOS) technology. Among the emerging paradigms, Ferroelectric Spin‐Orbit (FESO) devices have attracted considerable attention [1], leveraging the coupling between ferroelectricity and spin‐orbit interaction to enable electrically tunable spin functionalities within a single material. This approach is based on the possibilities offered by spin‐orbitronics [2], which exploits spin‐orbit coupling in non‐magnetic systems to generate charge currents from spin currents or vice versa. A particularly compelling material for such applications is germanium telluride (GeTe), which stands as the prototype of Ferroelectric Rashba Semiconductors (FERSCs) [3]. GeTe exhibits a non‐centrosymmetric rhombohedral ferroelectric *α*‐phase (space group *R3m*) at room temperature, transforming reversibly to a paraelectric cubic *β*‐phase (rocksalt structure) around 400°C [4, 5]. The *α*‐phase can be described as a distorted rocksalt structure elongated along one of the diagonals of the cubic unit cell of the *β*‐phase. This distortion arises from a relative displacement of the Ge and Te sublattices, leading to an alternation between short (2.84 Å) and long (3.16 Å) Ge─Te bond lengths [4]. This Peierls distortion, attributed to a particular chemical bonding mechanism coined as Metavalent bonding [6, 7], breaks inversion symmetry, giving rise to ferroelectricity. The polarization is aligned along the stretched diagonal and can point in either direction, resulting in eight possible ferroelectric domain orientations [8, 9, 10]. In addition to its ferroelectric properties, *α*‐GeTe exhibits a giant bulk Rashba‐type spin splitting [11, 12, 13]. Both theoretical and experimental studies have demonstrated that reversing the ferroelectric polarization switches the spin texture, making *α*‐GeTe thin films a promising material for the manipulation of spin and charge currents at room temperature in spintronic devices [11, 14, 15, 16, 17]. To date, proof‐of‐concept demonstrations have been carried out exclusively using quasi‐single‐crystal *α*‐GeTe(111) films exhibiting out‐of‐plane ferroelectric polarization obtained by molecular beam epitaxy (MBE).

While MBE enables the growth of *α*‐GeTe(111) films with excellent crystalline quality, [18, 19] its low deposition rate and the need for a Si(111)‐(√3 × √3)R30°‐Sb reconstructed substrate's surface, formed by annealing at around 700°C, significantly restricts scalability and impedes integration into microelectronics. Therefore, establishing a CMOS‐compatible and scalable fabrication method, such as industrial magnetron sputtering, is crucial for advancing beyond proof‐of‐concept devices to large‐scale development. This technique has already been used to produce high‐quality Sb_2_Te_3_ and GeTe/Sb_2_Te_3_ superlattices (SLs) films [20, 21, 22, 23, 24, 25, 26]. In the case of the SLs, initiating growth with a few nanometers of Sb_2_Te_3_ has been shown to be essential for achieving high structural quality [20, 21, 22, 23, 24, 25, 26]. Sb_2_Te_3_ is a layered material that crystallizes in a rhombohedral phase and forms quintuple layers (QLs) blocks of Te─Sb─Te─Sb─Te planes separated by *pseudo*‐van der Waals (vdW) gaps [27, 28]. In previous works, we have shown that vdW epitaxy enables the growth of oriented Sb_2_Te_3_ films by magnetron sputtering, with Sb and Te atomic planes parallel to the growth surface. Furthermore, this approach is effective across a wide range of substrates, facilitated by Te‐induced passivation of the substrate surface [23, 28]. Notably, first spin‐charge conversion results have been reported in Sb_2_Te_3_ films deposited by sputtering, confirming the potential of such films for spin‐based devices [29]. The results obtained for SLs indicate that the use of a Sb_2_Te_3_ seed layer may favor the growth of high‐quality *α*‐GeTe films by sputtering, suggested in the context of pulsed laser deposition (PLD) too [30]. To date, the deposition of high‐quality ferroelectric *α*‐GeTe(111) films by sputtering remains unexplored.

In this study, we investigate the structural and ferroelectric properties of *α*‐GeTe thin films, with thicknesses ranging from 25 to 100 nm, deposited by industrial magnetron sputtering onto a variety of substrates (amorphous a‐Si, thermal SiO_2_, and TiN, the latter commonly used as an electrode in devices). Prior to *α*‐GeTe growth, a 5 nm‐thick Sb_2_Te_3_ seed layer was deposited. Laboratory X‐ray diffraction and crystal orientation maps obtained from precession electron diffraction mapping reveal that *α*‐GeTe(111) films with polarization perpendicular to the surface can be successfully grown regardless of the substrate. The films exhibit a fiber texture with lateral crystallite sizes of approximately 30 nm. The Sb_2_Te_3_ seed layer is found to play a central role in determining the crystalline quality of the *α*‐GeTe films, as its structural properties govern those of the overlying *α*‐GeTe. High‐angle annular dark‐field scanning transmission electron microscopy images acquired within a crystallite show that the displacement of Ge atoms is uniform across the entire film thickness, indicating a ferroelectric polarization pointing outward the film surface. Simulation of anomalous X‐ray diffraction measurements performed around the Ge absorption K‐edge using synchrotron radiation further confirms that this polarization uniformity extends throughout the film. Finally, the reversal and control of polarization under an applied electric field is demonstrated by piezoresponse force microscopy.

## 2 Results

### 2.1 The Key Role of the Sb_2_Te_3_ Seed Layer on the Growth of Highly *c*‐Oriented *α*‐GeTe Film

Information on the deposition conditions of the GeTe films by magnetron sputtering on 200 and 300 mm diameter Si wafers are given in the Experimental Section. Prior to the growth of GeTe films, a 5 nm‐thick stoichiometric Sb_2_Te_3_ seed layer was grown. The formation of a vdW gap at the interface between the substrate and the first Sb_2_Te_3_ QL allows to grow Sb_2_Te_3_ on various types of substrate by vdW epitaxy [23, 28]. Seeded‐GeTe films were deposited on SiO_2_, amorphous Si (a‐Si) and TiN. In the following, the substrate name refers to the base substrate of the complete stack, even though the seeded‐GeTe film is always deposited on a Sb_2_Te_3_ layer.

**FIGURE 1:**
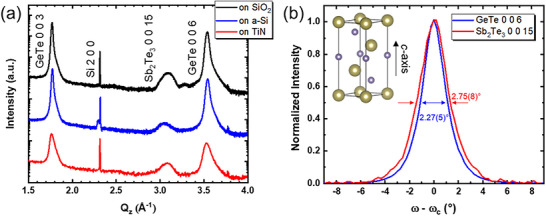
(a) Out‐of‐plane X‐ray diffraction patterns (*λ* = 1.5406 Å, Cu *Kα_1_* radiation), acquired in the *θ*/2*θ* geometry, as a function of the scattering vector *Q_z_* = 4π × sin(*θ*)/*λ*, for GeTe films with a 5 nm‐Sb_2_Te_3_ seed layer deposited on SiO_2_, a‐Si and TiN substrates. The GeTe and Sb_2_Te_3_ diffraction peaks are indexed as 0 0 *l* peaks (hexagonal indexation of their rhombohedral phases). The curves have been shifted vertically for clarity. (b) Rocking curves (*ω* scans, with *ω* the X‐ray incident angle, measured at a fixed 2𝜃 value corresponding to the maximum of the diffraction peak in a)) of the GeTe 0 0 6 and Sb_2_Te_3_ 0 0 15 reflections for the film grown on SiO_2_. Each rocking curve has been normalized (maximum intensity set to 1) and drawn as a function of *ω‐ω_c_*, with *ω_c_* the value of *ω* for the maximum of the curve. The hexagonal unit cell of *α*‐GeTe is shown as an inset.

The out‐of‐plane X‐ray diffraction pattern (XRD) of a 100 nm‐thick seeded‐GeTe film deposited on SiO_2_ is presented in Figure 1a. All diffraction peaks can be indexed as 0 0 *l* peaks (hexagonal indexation) of the GeTe and Sb_2_Te_3_ rhombohedral phases. As described in Figure S1, the hexagonal unit cell provides an intuitive description of the structure. Indeed, its *c*‐axis, aligned with ferroelectric polarization, is perpendicular to the film when Ge and Te planes are parallel to the substrate and Ge atoms displaced along the normal direction (so‐called *c*‐oriented film). As described in Supporting Information S1: Section 1, a *c*‐oriented film, the polarization is pointing upward (downward) if the Ge displacement *d_Ge_* is positive (negative). The detection of a Laue oscillation at *Q_z_* ​= 3.272 Å^−1^ around the 0 0 15 Sb_2_Te_3_ diffraction peak indicates the high structural quality of the seed layer. The lattice parameters for the seeded‐GeTe film deposited on SiO_2_ are *c_h_* = 10.664(4) Å and *a_h_* = 4.179(1) Å, closely matching those of *α*‐GeTe films grown by MBE on surface‐reconstructed Si (*c_h_* = 10.649 Å and *a_h_* = 4.17 Å) [18]. The in‐plane XRD pattern used to determine the *a_h_* value is presented in Figure S2. No preferred in‐plane crystallographic orientation is observed, as evidenced by the constant intensity measured during a *Φ* scan (rotation axis along the normal to the film) on the 1 1 0 Bragg diffraction peak (Figure S2), indicating a fiber texture. This is consistent with our previous reports on Sb_2_Te_3_ and GeTe/Sb_2_Te_3_ superlattice films grown using the same deposition technique [21, 23, 28, 31].

**FIGURE 2:**
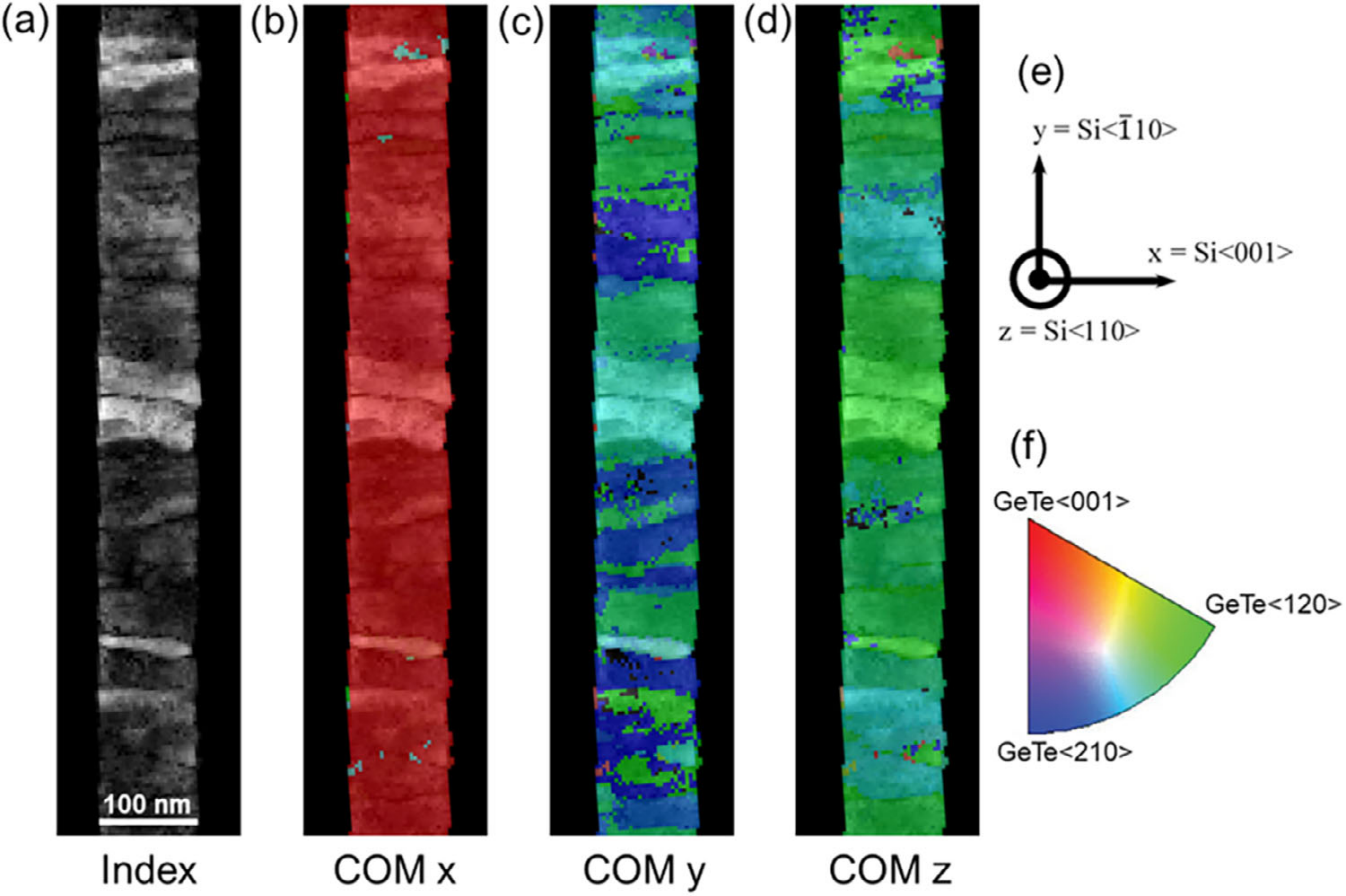
(a) Index map and (b–d) crystal orientation maps (COM), superimposed on the index one, along the *x*, *y*, and *z* directions respectively in the 100 nm‐thick Sb_2_Te_3_‐seeded GeTe film grown on SiO_2_ substrate. These axes are chosen with respect to the silicon substrate orientation as represented in (e). (f) Color legend used to visualize the orientation of the crystallites in the COM maps, using hexagonal indexation of *α*‐GeTe. The grey levels in the index map represent the correlation index between the simulated and measured electron diffraction patterns, with brighter areas indicating stronger matches.

To obtain a deeper insight into the texture of the films, crystal orientation maps (COM) were acquired using precession electron diffraction (PED) mapping. This technique enables the determination of the orientation of GeTe crystallites with a spatial resolution of about 2 nm. Figure 2 presents COM projected along directions related to the Si substrate as shown in Figure 2e. The maps reveal that the film is composed of crystallites with *c*‐axis oriented nearly perpendicular to the substrate surface and randomly varying in‐plane orientations, consistent with XRD patterns and highlighting a fiber‐textured structure. Most crystallites exhibit a vertical size comparable to the film thickness, while their lateral size ranges about 30 nm. A narrow tilt distribution of the *c*‐axis with respect to the film normal is present. It can be quantified using XRD from the analysis of rocking curves (*ω*‐scans), as illustrated in Figure 1b. The rocking curves of the GeTe 0 0 6 and Sb_2_Te_3_ 0 0 15 reflections have been normalized (maximum intensity set to 1) and plotted as a function of *ω‐Uω_c_*, with *ω_c_* the value of *ω* for the maximum of the curve. The raw data are provided in Figure S3. The full width at half maximum (FWHM) value of GeTe rocking curve is 2.27(5)° (instrumental contribution about 0.1°). As the width of the rocking curve is mainly related to the distribution of the *c*‐axis orientation with respect to the substrate normal, it evidences that the columnar crystallites are well‐oriented within the film. Strikingly, the FWHM value of the GeTe rocking curve closely matches that of the 0 0 15 reflection of the Sb_2_Te_3_ seed layer (2.75(8)°), indicating that the orientation of GeTe crystallites is driven by that of the Sb_2_Te_3_ crystallites. The critical role of the Sb_2_Te_3_ seed layer is confirmed by a comparison between seeded and unseeded GeTe films deposited on SiO_2_. While both films exhibit a *c*‐oriented growth, showing only GeTe 0 0 *l* reflections in out‐of‐plane diffraction, the FWHM of the 0 0 6 rocking curve in the unseeded GeTe film is 2.5 times larger than that of the seeded one (Figure S4). Furthermore, *θ*–2*θ* scans performed with an *ω* offset of 10° reveal diffracting GeTe crystallites in the unseeded film, where 0 0 *l* Bragg diffraction peaks are observed, a feature absent in the seeded one. These results clearly demonstrate that, in the absence of the Sb_2_Te_3_ seed layer, *α*‐GeTe film contains a significant fraction of misoriented grains. This also highlights that *θ*–2*θ* scans alone do not provide a definitive and comprehensive characterization of thin films.

For large‐scale integration, *α*‐GeTe thin films must be deposited on substrates other than SiO_2_. We investigated the growth of seeded GeTe on a‐Si and TiN, the latter being commonly used as bottom electrode in devices. The film thicknesses were set to 100 nm on a‐Si and 25 nm on TiN to match the typical dimensions in devices. Remarkably, high‐quality *c*‐oriented *α*‐GeTe(111) films can be successfully deposited regardless of the underlying material. The positions of the 0 0 3 and 0 0 6 GeTe diffraction peaks are almost the same for the various substrates, as illustrated in Figure 1a. The same conclusion applies for the 1 1 0 and 3 0 0 peaks detected in in‐plane XRD (Figure S2). Moreover, *Φ*‐scan measurements reveal a constant intensity, showing that the films possess a fiber texture with no preferential in‐plane orientation. The lattice parameters are *a_h_* = 4.1786(10) Å and *c_h_* = 10.662(4) Å for the seeded film grown on a‐Si and *a_h_* = 4.1765(15) Å and *c_h_* = 10.680(10) Å for that grown on TiN. These values are very close to the lattice parameters of the film grown on SiO_2,_ showing that the structure of GeTe is barely affected by the choice of the substrate. The FWHM of the rocking curve of the *α*‐GeTe 0 0 6 reflection is 3.50(6)° on a‐Si and 3.90(6)° on TiN. For each film, these values are close to the FWHM of the rocking curve the Sb_2_Te_3_ 0 0 15 reflection, as illustrated for the sample grown on TiN in Figure S3. This confirms that the *α*‐GeTe films inherit the *c*‐axis tilt distribution from the Sb_2_Te_3_ seed layer, which is slightly broader for deposition on a‐Si or TiN than for deposition on SiO_2_. The resulting rocking curve broadening reduces the diffraction peak intensity as visible in Figure 1a. Given identical deposition conditions for both the seed and GeTe layers, the variations in tilt distribution are most likely driven by substrate roughness, which directly impacts the crystalline quality of the Sb_2_Te_3_ seed layer.

At this stage, X‐ray diffraction analysis shows that the use of a Sb_2_Te_3_ seed layer grown by vdW epitaxy enables the successful growth of *α*‐GeTe(111) thin films by sputtering on various substrates, a crucial step towards large‐scale integration in devices. The films display a fiber texture with a lateral crystallite size of approximately 30 nm and are *c*‐oriented, implying that Ge atoms are displaced along the film normal, which gives rise to an out‐of‐plane ferroelectric polarization. In order to obtain information on the direction of atomic displacement of Ge atoms and, consequently, on the upward or downward orientation of polarization, scanning transmission electron microscopy and anomalous X‐ray diffraction analyses were performed, as described in the following sections.

### 2.2 Evidence of Uniformly Polarized Crystallites

**FIGURE 3:**
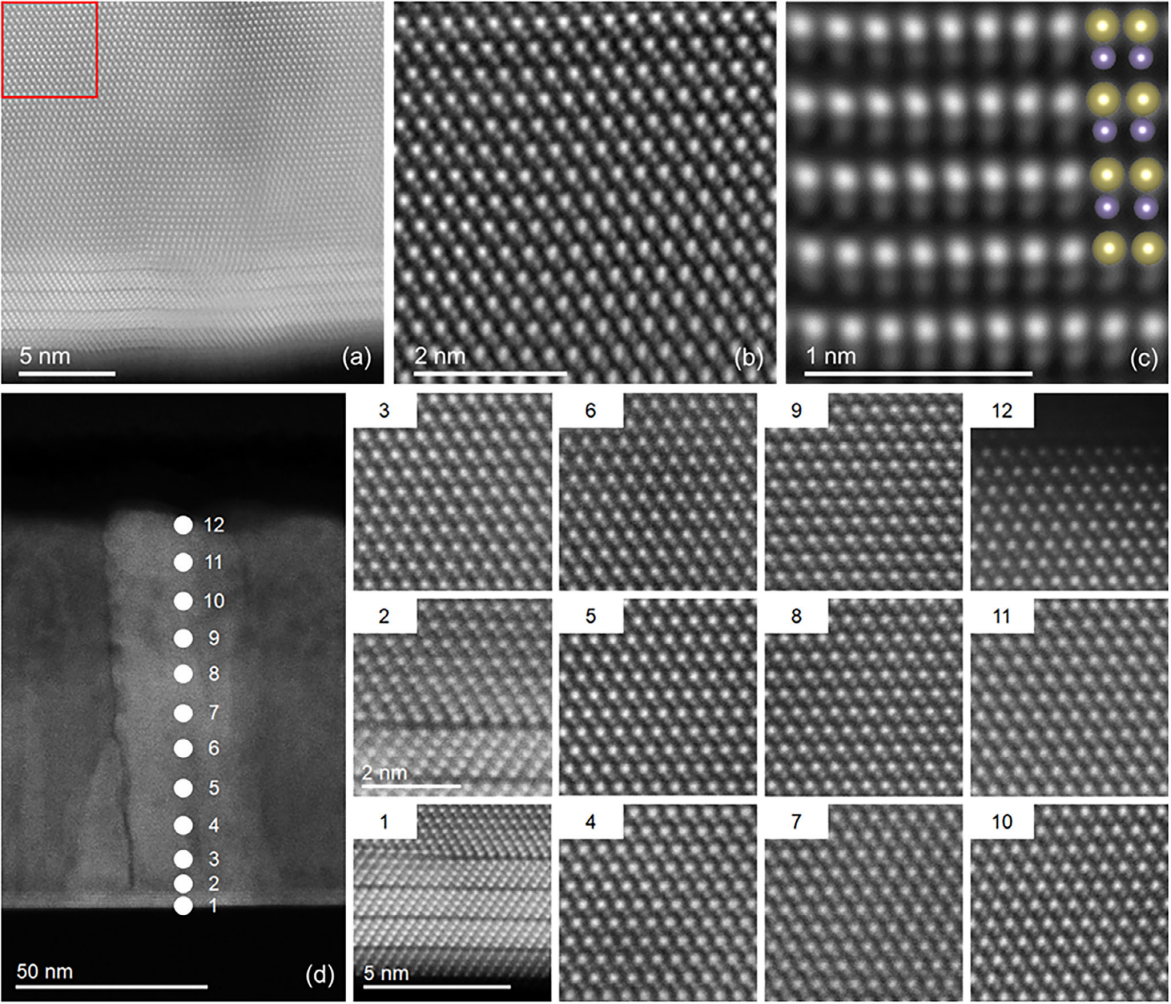
(a) HAADF‐STEM image of a Sb_2_Te_3_ seeded GeTe film grown on SiO_2_. The Sb_2_Te_3_ QLs of the seed layer above the substrate can be easily distinguished at the bottom of the image. (b) Zoom in the 5 × 5 nm^2^ red square plotted in image (a) highlighting non‐equidistant Ge and Te planes parallel to the film surface. Viewing direction along the zone axis <1 1 0>. (c) Enlarged view of another GeTe grain with a different in‐plane orientation than the one shown in (b). Ge and Te columns are highlighted by purple and yellow spheres, respectively. Viewing direction along the zone axis <2 1 0>. For images (b) and (c), denoising was carried out using a classical Fourier‐domain filtering approach, where noise‐related frequency components were selectively masked, followed by image reconstruction via inverse Fourier transformation. (d) Twelve enlarged views acquired in a GeTe crystallite across the entire film thickness from the Sb_2_Te_3_ seed layer to the amorphous SiN_x_ capping layer. The positions of acquisition of these images are depicted with white disks in the overall view displayed in (d). Pictures 2 to 12 have the same size. Image 1 has a lower magnification in order to evidence the Sb_2_Te_3_ seed layer and its interfaces.

High‐angle annular dark‐field scanning transmission electron microscopy (HAADF‐STEM) was used to further investigate the structure of the Sb_2_Te_3_‐seeded GeTe film grown on SiO_2_. While X‐ray diffraction and precession electron diffraction provide comprehensive information on the orientation, size, and morphology of crystallites, the Z‐contrast of HAADF‐STEM images allows a clear distinction between light (Ge) and heavy elements (Te and Sb). Thus, this enables direct visualization of local atomic ordering with a high spatial resolution [32]. The magnification in Figure 3a was selected to visualize the stacking of GeTe on the Sb_2_Te_3_ seed layer and the interface of the latter with the SiO_2_ substrate. Four Sb_2_Te_3_ QLs are clearly observed, indicating that intermixing occurs between the last QL and the bottom of the GeTe layer during growth. A magnification of the region marked with a red square in Figure 3a is shown in Figure 3b. The latter reveals the parallel arrangement of non‐equidistant Ge and Te atomic planes and confirms the *c*‐oriented stacking of *α*‐GeTe. The atomic columns are well‐defined as observed in GeTe crystals [33], further attesting to the high structural quality of the film. Within the area probed by STEM in Figure 3b, the Ge planes are shifted upward. Figure 3c displays another high‐resolution HAADF‐STEM image acquired from a crystallite oriented along a different zone axis. Here again the Ge displacement is upward as highlighted by the observation of long and short projected Ge─Te distances in the vertical direction (see Supporting Information S1: Section 1). These distances, averaged over the entire image, yield to an estimated lattice parameter *c_h_* of 10.53 Å. This value is consistent with the XRD value of 10.66 Å within the experimental uncertainty of TEM analysis, which may result from various effects: thin‐foil preparation and relaxation, as well as distortions introduced by sample drift, vibration, or denoising procedures.

The images presented in Figure 3b,c show that Ge atoms are displaced upward, but this information is limited to a small region and does not provide a complete picture of the ferroelectric polarization across the film thickness. Given that the crystallites span the full film thickness, we performed STEM imaging along the *c*‐axis direction of a crystallite in order to directly assess the polarization profile across its entire height. Thus, one crystallite was randomly selected, and twelve high‐resolution HAADF‐STEM images were acquired from the substrate to the top surface (Figure 3d). The magnification of image 1 showing the interface with the substrate is lower than that of the other images in order to highlight the high quality of the Sb_2_Te_3_ layer. Four Sb_2_Te_3_ QLs separated by *pseudo*‐vdW gaps are clearly visible. The gap located above the fourth QL is associated with a 60° in‐plane rotation (twin). It can be noticed that the fifth Sb_2_Te_3_ QL intermixes with the first GeTe atomic planes. The second image shows the interface of GeTe with the seed layer. The 12th image shows the upper part of the film, covered by the amorphous SiN_x_ capping layer. The top of the film is not flat (Figure 3d). All other images were acquired within the GeTe film, and enlarged views extracted from the regions marked with white discs in the whole crystallite image are shown in Figure 3d. In all of these images, the Ge atomic columns are displaced toward the top. This shows that the polarization is oriented upward across the entire thickness of the crystallite and hence of the film. Therefore, this indicates also that no other ferroelectric domains exist in the crystallite studied, the latter being uniformly polarized.

To gain deeper insight into the intermixed transition between the Sb_2_Te_3_ seed layer and the GeTe film, linear intensity profiles were extracted. The results are provided in Figure S7. Within the intermixed region, a progressive change of HAADF signal from high intensity peaks, corresponding to pure Sb columns, to weak intensity ones, corresponding to pure Ge columns, is observed. This clearly indicates a gradient of local intermixing of Sb and Ge atoms on the Ge/Sb sublattice positions extending over about 2‐3 nm. It should be noted that the transition between the pure Sb_2_Te_3_ seed layer and the GeTe film is smooth, without the formation of a Te─Te vdW‐gap at the transition between both materials. This transition rather occurs through a gradual enrichment in Ge of Sb cationic planes forming intermixed Ge/Sb planes, alternatively with the Te pure anionic planes, during the transition from Sb_2_Te_3_ to GeTe.

### 2.3 Evidence of Uniformly Polarized State in the Whole Film

The above high‐resolution transmission electron microscopy study clearly demonstrates that the displacement of Ge atoms, and therefore ferroelectric polarization, is uniform and directed upward in the studied crystallite. However, only a few grains can be studied by TEM. Nevertheless, the polarization orientation at the macroscopic scale can be studied by performing X‐ray diffraction experiments at different X‐ray energies close to that of the Ge absorption K‐edge, as shown below and in reference [34]. The integrated intensity of a diffraction peak is proportional to the square of the modulus of the unit cell structure factor. For X‐ray energies close to an absorption edge, dispersion (anomalous) real and imaginary contributions to the atomic scattering factor of the selected atom must be included, and the unit cell structure factor strongly depends on the X‐ray energy [35, 36]. Besides, in non‐centrosymmetric structures, Friedel's law is broken, meaning that the diffracted intensity for *h* *k* *l*  and $\bar{h}\;\bar{k}\;\bar{l}\;$ reflections may differ. X‐ray diffraction at energies close to an absorption edge, so called anomalous diffraction, can thus be used in polar crystals to determine the direction and sense of atomic displacements [34, 37, 38].

**FIGURE 4:**
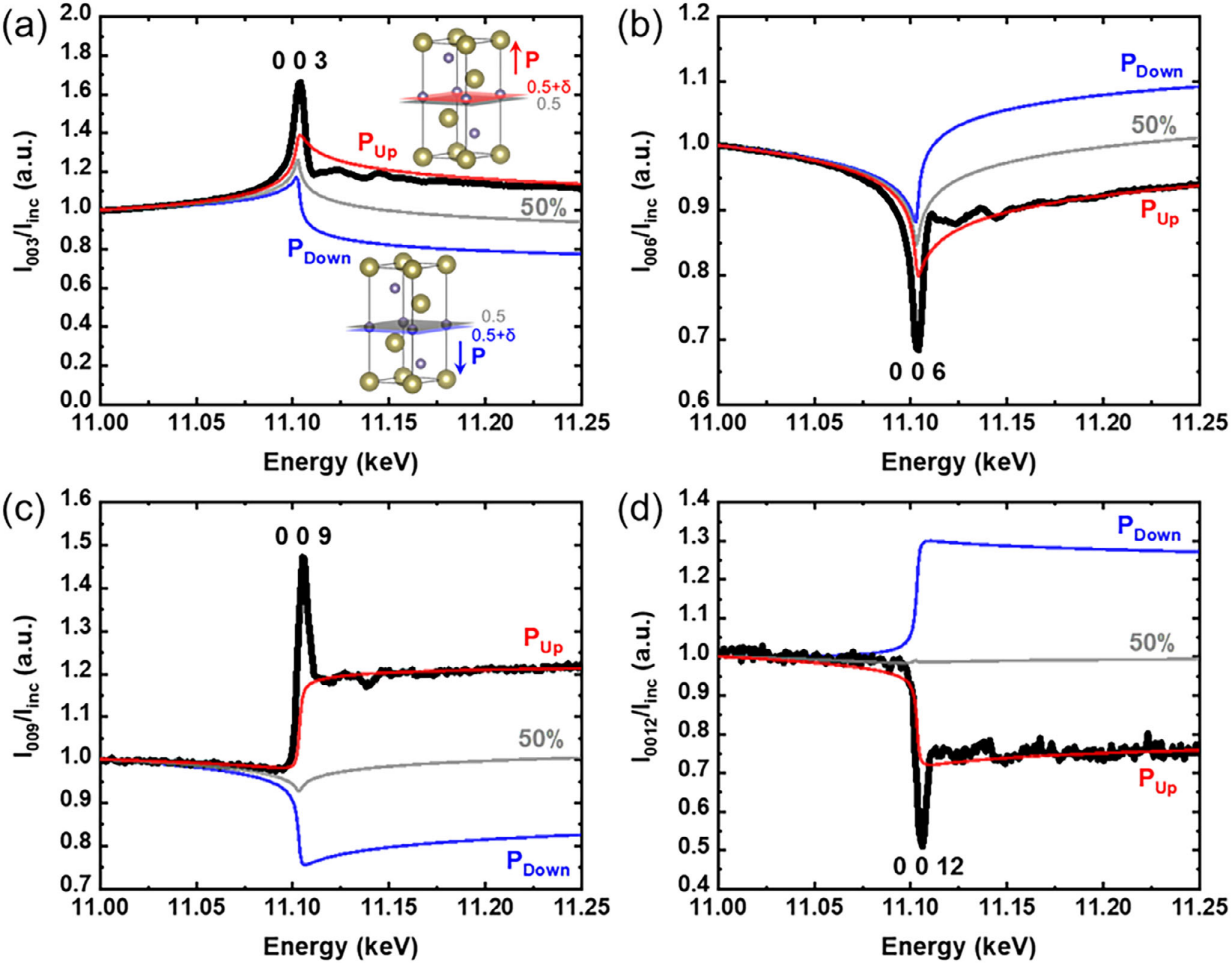
Integrated intensities for 0 0 *l* diffraction peaks, divided by the incident beam intensity *I_inc_* and normalized to 1 for *E* =11 keV (black curves), for the 100 nm‐ thick seeded GeTe film deposited on SiO_2_. Calculated intensities, normalized to 1 for *E* =11 keV, are drawn assuming a film entirely polarized upward (*P_Up_*, red curves), entirely polarized downward (*P_Down_*, blue curves) and a film without macroscopic polarization containing equal volumes of ferroelectric domains oriented in one direction or the other (50%, grey curves). The peak observed for *E* ≈  *E*_0_ is due to the near‐edge fine structure. Calculations were performed for  *δ*=+0.025 or ‐0.025. The Ge relative coordinate in the hexagonal unit cell is 0.5 + *δ*. A positive δ value corresponds to a polarization pointing upward and a negative one to a polarization pointing downward, as shown in the inset in (a).

The integrated intensity *I*_00*l*_(*E*) of 0 0 *l* Bragg diffraction peaks (*l* = 3, 6, 9, 12) was measured as a function of the X‐ray energy *E* in the range [11–11.25 keV] around the energy *E*_0_ =  11.1062 keV of the Ge K‐edge (see the Experimental Section) for the 100 nm‐thick seeded‐GeTe film deposited on SiO_2_. In the experimental conditions used (beam size ≈ 0.16 mm in the scattering plane and ≈ 0.26 mm in the perpendicular direction), the illuminated surface varies from 0.26 mm^2^ for *l* = 3 to 0.066 mm^2^ for *l* = 12. Its decrease as *l* increases reflects the decrease in the footprint of the incident beam as the angle *ω* between the incident beam and the film surface increases (ω ≈ 9° for *l* = 3 and ω ≈ 39° for *l* = 12). The penetration length of X‐rays in GeTe (20 µm for *E* < *E*_0_ and 11 µm for  *E* > *E*_0_) is much larger than the film thickness. Whatever the energy and the *l* value, the entire film thickness is therefore probed. The energy dependence of *I*_00*l*_(*E*)/*I_inc_* (*E*), with *I_inc_* (*E*) the intensity of the incident beam, reveals that of the square modulus of the unit cell structure factor |*F*_00*l*_(*E*)|^2^ (see the Experimental Section). Above the edge, *I*_00*l*_(*E*)/*I_inc_* (*E*) is the sum of a smooth contribution that varies slowly with *E*, and a contribution that oscillates rapidly with *E* (Figure 4). The latter is the so‐called diffraction anomalous fine structure, which contains structural information on the local environment of Ge atoms. The change in the smooth part, when *E* increases across the energy edge, contains the requested information about the polarization state in the GeTe film. Analyzing the fine structure is out of the scope of the present work but when comparing the values of *I*_00*l*_(*E*)/*I_inc_* (*E*) measured for *E* smaller than *E*_0_ and *E* larger than ≈ 11.15 keV, where the fine structure contribution is reduced, striking differences are observed according to the *l* value. The intensity increases for *l* = 3 and 9 and decreases for *l* = 6 and 12. The measured intensities are compared in Figure 4 with calculated ones obtained by multiplying the calculated |*F*_00*l*_(*E*)|^2^ values by geometrical and absorption terms and then normalizing to 1 for *E* = 11 keV (see the Experimental Section). The unit cell structure factor was calculated assuming either a film that is entirely polarized upward |*F*_00*l* *Up*_(*E*)|^2^ or entirely polarized downward |*F*_00*l* *Down* _(*E*)|^2^. In addition, the intensity expected for a film containing equal volumes of ferroelectric domains oriented in one direction or the opposite, thus with no macroscopic polarization, was obtained by calculating (|*F*_00*l* *uUp*_(*E*)|^2^ + |*F*_00*l* *Down* _(*E*)|^2^)/2, assuming that X‐ray scattering by the two kinds of ferroelectric domains is incoherent. |*F*_00*l* *Up*_(*E*)|^2^ and |*F*_00*l* *Down* _(*E*)|^2^ are significantly different for *E* > *E*_0_. Moreover, the difference |*F*_00*l* *Up*_(*E*)|^2^ − |*F*_00*l* *Down* _(*E*)|^2^ is positive if *l* is odd and negative if *l* is even. For all *l* values, the experimental intensities are in very good agreement with calculations assuming a film entirely polarized upward. For the calculations shown in Figure 4, the relative position of Ge atoms in the hexagonal unit cell is 0.5 + *δ* with *δ* = 0.025. This *δ* value leads to the best agreement with experimental data as shown in Figure S9, where simulations for different *δ* values are presented. It is also in agreement with the value deduced from neutron diffraction on a GeTe single crystal as obtained in reference [4]. Ge atoms are thus displaced upward by *d_Ge_* = *δ* × *c_h_* = 0.267 Å in the studied GeTe film. It was not necessary to include the effect of atomic vibrations (Debye‐Waller DW factors) in the calculations shown in Figure 4. At room temperature, the DW factors for Ge and Te reported in the literature for single‐crystal GeTe [4] or polycrystalline GeTe [5] are very similar. Thus, |*F*_00*l*_(*E*)|^2^  values calculated taking DW factors into account differ from those that neglect them only by a multiplicative factor that depends on *l*. This has no effect on Figure 4, where the experimental and calculated intensities have been independently normalized to 1 for each value of *l*.

To summarize, the comparison between the experimental and calculated intensities allows us to conclude that the polarization in the GeTe film studied, deposited by sputtering on a 200/300 mm diameter wafer, is uniform and directed upward. The high sensitivity of anomalous X‐ray diffraction around the absorption edge of Ge to the polarization state of a GeTe film can be seen in Figure 4 by comparing the experimental intensities with those calculated for an unpolarized film or a downward‐polarized film. It must be emphasized that the volume probed by X‐rays is macroscopic (illuminated surface of the order of 0.1 mm^2^ and penetration length of X‐rays much larger than the film thickness).

### 2.4 Electrical Control of Ferroelectric Domain Polarization

The results presented above show that high‐quality and uniformly upward polarized films have been successfully deposited using an industrially scalable technique. To fully validate the suitability of these films for practical applications in devices, it is essential to investigate whether their ferroelectric polarization can be reliably controlled and reversed.

**FIGURE 5:**
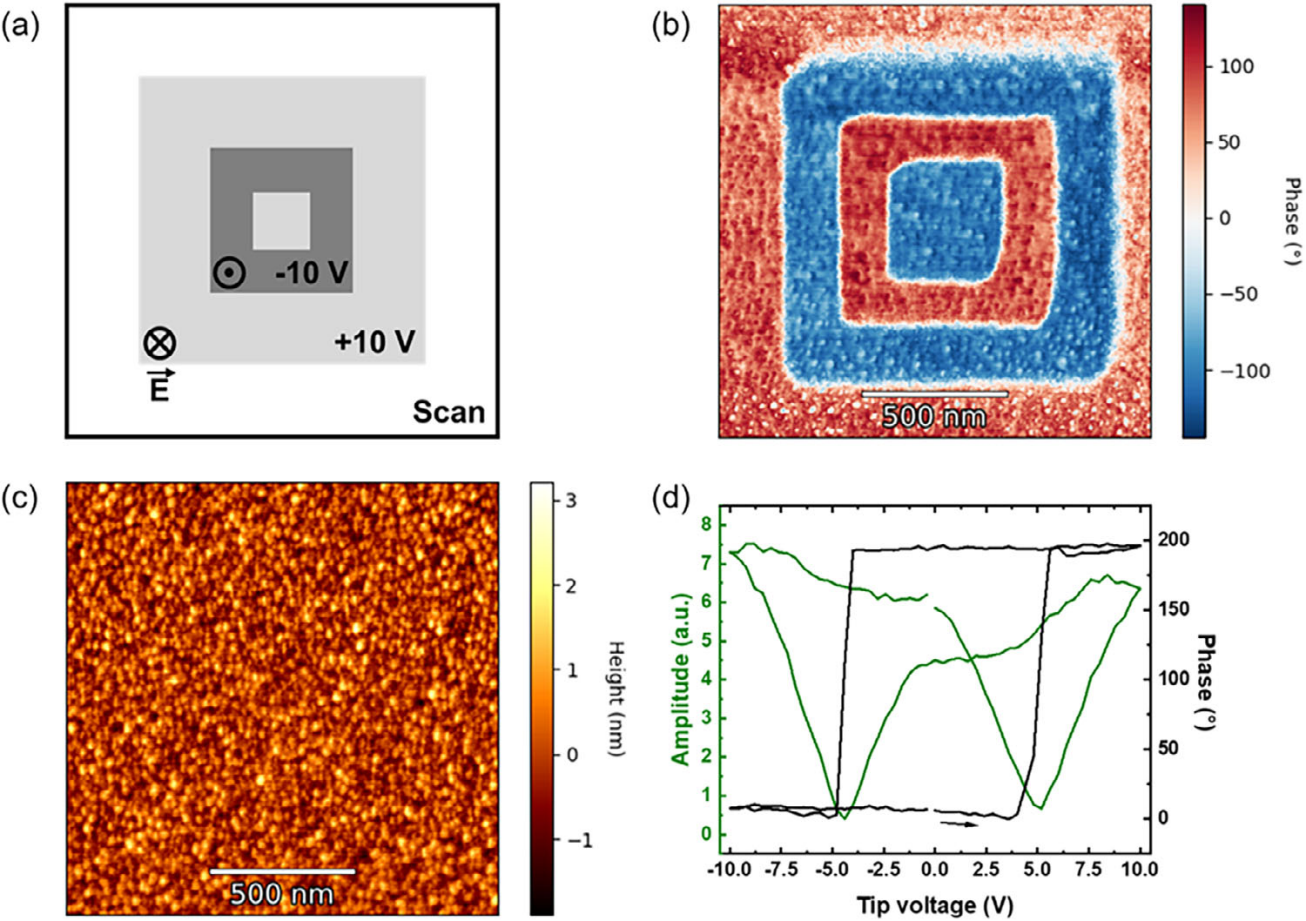
Vertical PFM characterization of a 40 nm‐thick seeded‐GeTe film grown on an a‐Si substrate and capped with a 10 nm thick SiN_x_ layer. (a) Schematic illustration of the writing procedure used to locally control ferroelectric polarization. When the tip voltage is positive (negative), the applied electric field E is downward (upward). (b) PFM phase image acquired in the DFRT mode after the writing process. (c) Surface topography measured by AFM. (d) Amplitude and phase hysteresis loops obtained with SSPFM‐DFRT mode. The curves are the average of ten acquisitions.

To evaluate the polarization switching capability by means of application of an electrical field, we performed vertical piezoresponse force microscopy (PFM) [39] on a 40 nm‐thick *α*‐GeTe film grown on a‐Si. In this technique, a conductive tip of an atomic force microscope (AFM) is brought into contact with the surface of the ferroelectric sample. A periodic voltage is applied through the tip, inducing localized piezoelectric deformations within the material, which can be measured and give information about the local ferroelectric state. Hence, by scanning the film surface, a ferroelectric domain mapping can be obtained. Information on the measurement procedure can be found in the Experimental Section. Prior to the measurement of the piezoelectric deformation, a DC voltage of ±10 V was applied to the tip during scanning of the film surface, as shown in Figure 5a, in order to control the polarization orientation. A square region (1 × 1 µm^2^) was first scanned by applying a positive voltage to the conductive tip. Subsequently, a smaller square (0.75 × 0.75 µm^2^) was written within the first region using a negative voltage. A final inner square was then scanned positively. PFM‐DFRT (dual‐frequency resonance tracking) phase and amplitude images were then acquired. The phase image displays clear ferroelectric domain patterns with 180° phase contrast between adjacent regions (Figure 5b). Indeed, the region scanned under an upward electric field exhibits the same phase (red contrast) as the pristine film area, whose anomalous diffraction measurements show that it is polarized upward. In contrast, the region written with a downward electric field shows a blue contrast, corresponding to a down‐polarized area, demonstrating that applying a ±10 V DC bias allows to pole the film. The granular aspect of the image is correlated with the film topography measured by AFM and shown in Figure 5c. The root mean square roughness (RMS) value is found to be 0.50 nm and the lateral grain size is estimated to about 30 nm, consistent with the crystal orientation maps acquired using precession electron diffraction shown in Figure 2a–d.

To further investigate the ferroelectric behavior of the *α*‐GeTe film, switching spectroscopy PFM (SSPFM) was performed. Observation of a piezoresponse hysteresis loop, averaged over 10 cycles (Figure 5d), demonstrates reversible polarization switching. The butterfly shape of the amplitude and the nearly symmetric phase loops exhibit signal inversion around ±5 V, consistent with values reported for a 40 nm‐thick GeTe film deposited by MBE and capped with amorphous SiN_x_ [17]. To confirm the functional validation of TiN as a device‐compatible bottom electrode, a 40 nm‐thick seeded‐GeTe film was deposited on a 10 nm TiN layer and studied by PFM. The ferroelectric switching behavior of the GeTe film grown on TiN closely resembles that of a GeTe film grown on a‐Si, confirming the robustness of the polarization reversal across different substrates. A higher tip voltage (∼±7.5 V) is required to switch the polarization on TiN compared to a‐Si (∼±5 V). Beyond the fact that the tips used for both measurements are not the same, this effect could be attributed to differences in electrical conductivity within the layer stack leading to changes in the electric field distribution. Additionally, tip–sample contact effects, potentially enhanced by increased surface roughness in the case of the film deposited on TiN, may limit full saturation of the PFM response. The deposition of a finite size TiN bottom electrode like in device configuration is expected to mitigate these effects. In the literature, voltages at which the PFM phase switches by 180° are sometimes incorrectly referred to as coercive voltages. This terminology can be misleading, as the capping layer attenuates the applied electric field, which is additionally intrinsically non‐uniform due to the tip geometry. As a result, the observed phase switching voltage in PFM measurements does not correspond to the intrinsic coercive field of the ferroelectric material, but rather reflects the behavior of the entire stack and gives an upper limit for the coercive value.

As PFM techniques are subject to numerous artefacts [40], especially when applied to conductive layers such as *α*‐GeTe [41, 42], control measurements were conducted using a SiN_x_ capped 40 nm‐thick Sb_2_Te_3_ layer that exhibits a similar electrical conductivity (*σ_GeTe_* ∼ 1.1 × 10^3^ S.cm^−1^ and *σ_Sb2Te3_* ∼ 1.6 × 10^3^ S.cm^−1^). The room‐temperature electrical conductivities were measured using the conventional van der Pauw method, with contacts placed at the sample corners. In the case of the Sb_2_Te_3_ layer, only charge injection behavior was observed (Figure S11). The absence of ferroelectric‐like switching in that sample supports the conclusion that the PFM signal measured in the GeTe film includes a ferroelectric contribution, although possibly superimposed with minor charge injection effects.

## 3 Discussion

We have demonstrated the successful deposition of ferroelectric *α*‐GeTe(111) thin films using industrial high‐vacuum magnetron sputtering at temperatures of approximately 250°C. The use of a Sb_2_Te_3_ seed layer prior to GeTe growth is the key parameter to obtain highly‐textured *α*‐GeTe(111) thin films with an out‐of‐plane ferroelectric polarization. The films exhibit a fiber texture, consisting of columnar GeTe crystallites that extend throughout the thickness of the film, with lateral dimensions ranging from 20 to 50 nm and no twin domains. The surface of the films exhibits a very low roughness of 0.50 nm RMS, with no traces of holes. Note that, even without any further process parameter optimization, an excellent deposition homogeneity over up to 300 mm‐diameter wafers could be obtained (see Figures S5 and S6). X‐ray diffraction shows that the *c*‐axis orientation of the *α*‐GeTe(111) films is maintained along the wafer's radius. Only a slight increase in the width of the rocking curve (of about 1°) is observed when moving away from the center toward the edge, most likely due to a slight temperature gradient across the wafer in the sputtering deposition tool. The use of a low‐temperature industrial process that is fully compatible with back‐end‐of‐the‐line CMOS integration (*T* < 450°C) represents a major advance over *α*‐GeTe(111) thin films developed by MBE on reconstructed Si substrates. In the case of MBE, a specific preparation of the substrate surface at a temperature above 450°C is mandatory prior to GeTe film growth. Furthermore, unlike our deposition technique, *α*‐GeTe(111) films obtained by MBE often exhibit undesirable discontinuities between coalesced islands [19]. The same conclusion applies to films deposited by pulsed laser deposition on Si(111) [30]. In this case, the use of 2D‐bonded Sb_2_Te_3_ seed layer promotes 2D growth of the GeTe film, thereby promoting the formation of flat and uniform layers.

A slight angular spread in the *c*‐axis orientation of the GeTe crystallites is present from 2.27(5)° (FWHM) for films deposited on SiO_2_ to 3.90(6)° for films deposited on TiN. The tilt of the GeTe crystallites corresponds to that of the Sb_2_Te_3_ crystallites of the seed layer. These results highlight the critical role of the Sb_2_Te_3_ seed layer in achieving high‐quality growth of ferroelectric *α*‐GeTe(111) thin films. This conclusion is also supported by the observation of misoriented GeTe crystallites in a GeTe film deposited directly on SiO_2_. The lattice parameters of the seeded GeTe films are almost independent of the nature of the substrate. This can be attributed to the vdW epitaxy of the Sb_2_Te_3_ seed layer, which accommodates various substrates due to the presence of an interfacial vdW gap [23, 28]. Given that the Sb_2_Te_3_ seed layer plays a pivotal role in the high‐quality growth of *α*‐GeTe(111) on different substrates, it is instructive to examine the nature of the interface between these two materials. At the Sb_2_Te_3_/GeTe interface, we observe a twin boundary and a region of chemical intermixing, suggesting local interdiffusion of the atomic species between both layers [21, 43]. Importantly, no Te─Te gap forms between the two layers, with a gradual transition between Sb_2_Te_3_ and GeTe thanks to the formation of an intermediate Ge─Sb─Te layer. This intermediate Ge─Sb─Te layer is composed of cationic Ge/Sb planes and pure anionic Te planes and is approximately 2–3 nm thick. This explains the correspondence between the out‐of‐plane orientation and the similarity between the XRD rocking curves between GeTe film and its Sb_2_Te_3_ seed layer.

Moreover, HAADF‐STEM and anomalous diffraction analysis show that the Ge planes are consistently shifted upward relative to the Te planes, evidencing uniform upward ferroelectric polarization across the 100 nm‐thick film. The same conclusion was drawn for a 200 nm‐thick film elaborated by MBE [18]. In a 60 nm‐thick MBE film, a few amount of domains with tilted ferroelectric polarization has been observed [44]. In the literature, angle‐resolved photoemission spectroscopy (ARPES) and PFM have been commonly employed to probe the polarization orientation in MBE‐grown GeTe films [12, 15]. While ARPES is highly sensitive to the ferroelectric polarization, since the observed Rashba spin splitting depends directly on the polarization state [11], its probing depth is limited to only a few nanometers, providing information restricted to the near‐surface region. Similarly, in PFM, the effective probing depth is poorly defined due to the tip–sample geometry [39]. In contrast, anomalous diffraction, with its inherent bulk sensitivity, uniquely enables determination of the polarization state across the entire GeTe film thickness and over a large area, offering direct insight into the macroscopic ferroelectric order. The absence of ferroelectric domains in our films contrasts with GeTe bulk crystals obtained through slow cooling from the liquid state. In such cases, the GeTe first solidifies in the cubic *β*‐phase and then transforms into the ferroelectric *α*‐phase at about 400°C [4], which can exhibit either inversion domains (about 15 nm‐thick in reference [33]), or a so‐called herringbone domain structure [9, 10]. For *c*‐oriented *α*‐GeTe films, the material is deposited at around 250°C, which means it crystallizes directly into its ferroelectric *α*‐phase.

Let us discuss in the following the possible reasons of the absence of domains in *c*‐oriented *α*‐GeTe films. First, the relative displacement between the Ge and Te sublattices generates a spontaneous polarization, but also a depolarizing field. In ferroelectric materials, the formation of domains with different polarization orientations minimizes the electrostatic energy. The fact that no ferroelectric domains are observed after deposition in the *α*‐GeTe film could be explained by the presence of free carriers. First‐principles calculations have highlighted the influence of free carriers to stabilize a ferroelectric phase in ultra‐thin GeTe films [45]. Although *α*‐GeTe is a narrow‐gap semiconductor, it has significant electrical conductivity due to *p*‐type self‐doping caused by a high density of intrinsic Ge vacancies [41, 42]. In a thin film, under the action of the depolarizing field, these free carriers migrate toward the two interfaces of the film, creating an opposing electric field that could screen the depolarizing field. Secondly, the critical role of strain has been proposed in the case of self‐poled PZT films [46]. A crystallographic texture or epitaxial growth of the PZT film permits to avoid random averaging of polarization directions. More importantly, a hierarchical heterogeneity along the film thickness (through gradients in composition, lattice strain, or defect‐induced charge) is used to generate an internal driving force that aligns the polarization. Phase‐field simulations confirm that a strain gradient across the ferroelectric thin film produces a strong internal field that orients the spontaneous polarization out of plane, whereas films without such gradients show random polarization patterns. In the *α*‐GeTe film studied here, a residual compressive stress of about 70 MPa is deduced from substrate curvature measurement. It results from the difference in linear thermal expansion coefficients between the film and its substrate. The induced strain is expected to be non‐constant along the film thickness and could be at the origin of the self‐polarization observed in our *α*‐GeTe films. Last, density functional theory studies have indicated that Te surface termination is energetically favored in *α*‐GeTe slabs [47]. The influence of *α*‐GeTe surface termination on polarization direction has been studied experimentally either in bulk single crystals [33] or in MBE grown films [15]. While Te‐terminated surfaces exhibit upward polarization as revealed by ARPES [15, 16] and PFM [12, 15, 16], Ge‐terminated surfaces are instead polarized downward. In our TEM images, the low contrast at the film surface prevents unambiguous identification of the terminal atomic species. Besides, we draw attention to the fact that, in the case of thin films, not only the upper surface of *α*‐GeTe but also the bottom interface should be considered. In order to rule out any influence of the Sb_2_Te_3_ seed layer on the self‐polarized behavior, we investigated a film grown without a Sb_2_Te_3_ seed layer. This film exhibits a high proportion of misoriented crystallites (see Figure S4). Anomalous X‐ray diffraction measurements performed on the 0 0 *l* reflections probe the *c*‐axis oriented GeTe crystallites. For this unseeded film, we also observe an upward self‐polarization (see Figure S10).

The clear hysteresis loops with both phase and amplitude PFM signals obtained in our *α*‐GeTe(111) thin films confirm a switchable ferroelectric polarization with reversal voltages about ±5 V. These results are in very good agreement with those reported for 40 nm‐thick MBE‐grown films capped with amorphous SiN_x_. In that case, PFM measurements also revealed a nearly centered hysteresis loop with a distinct phase reversal for same tip tension. These results are further supported by resistive modulation experiments in Ti/GeTe junctions [17]. Since the seeded *α*‐GeTe(111) sputtered films exhibit ferroelectric properties comparable to those of MBE‐grown films that have already been integrated and successfully employed as proof‐of‐concept devices, these results highlight the strong potential of the sputtering‐based growth approach developed in this work.

## 4 Conclusion

We have established industrial magnetron sputtering as a CMOS‐compatible technique for the scalable growth of ferroelectric *α*‐GeTe(111) thin films, a promising material for room‐temperature spin‐orbitronics. The use of a thin Sb_2_Te_3_ seed layer is found crucial to achieve high‐quality films as it reduces crystallite misorientations. This seed layer being grown by vdW epitaxy, high quality *α*‐GeTe(111) films can be obtained on a variety of technologically relevant substrates, overcoming the limitations of other deposition techniques. The sputtered films match the ferroelectric properties of MBE‐grown films that have been integrated for proof of concept. The films exhibit a uniform polarization at the macroscopic scale and the latter can be electrically switched with low voltages (about 5 V), a key point for devices. By making the ferroelectricity and strong Rashba spin splitting of *α*‐GeTe(111) compatible with the mainstream microelectronics industry, this study mitigates an important limitation in the development of Rashba ferroelectric semiconductors for spin–orbit devices, with potential implications for low‐power spin memory and logic.

## 5 Experimental Section

### 5.1 Thin‐Film Deposition

GeTe films were deposited using industrial high‐vacuum magnetron sputtering equipment. Two distinct sets of samples, fabricated on either 200 mm or 300 mm Si(100) wafers using two different sputtering tools, were investigated. The selected substrates for this study are thermal SiO_2_, amorphous Si and TiN. The amorphous Si substrates were obtained by Ar‐plasma cleaning of the surface of Si(001) wafers to remove the nm‐thick native oxide, resulting in the formation of a ∼2 nm thick a‐Si layer on top of the monocrystalline Si substrate. TiN substrates were fabricated by depositing a 10 nm‐thick polycrystalline TiN layer on thermal SiO_2_ substrates. Prior to GeTe deposition, a 5 nm‐thick Sb_2_Te_3_ seed layer was grown. Growth conditions were optimized using 100 nm‐thick Sb_2_Te_3_ films to ensure high‐quality seed layer deposition, with substrate temperatures set to either 200°C or 250°C, depending on the sputtering system used. It should be noted that the real substrate temperature may slightly deviate from the nominal setpoint. At these temperatures, sputtering from a stoichiometric Sb_2_Te_3_ target results in Te‐deficient films [21, 24]. Therefore, based on our previous works, the seed layers were deposited by co‐sputtering from a stochiometric Sb_2_Te_3_ target and an additional pure Te target [21, 23]. Under these Te‐rich conditions, highly oriented Sb_2_Te_3_ layers grow on a variety of substrates due to the formation of a Te‐enriched interfacial layer and a vdW‐gap between the substrate and the first Sb_2_Te_3_ quintuple layer [23, 28]. Following the seed layer deposition, GeTe films with nominal thickness in the range of 25‐100 nm were obtained by sputtering a GeTe target. For comparison, unseeded GeTe films were also deposited from the same target. In the absence of a seed layer, the optimal growth temperature is 300°C instead of 250°C with a seed layer. Film thicknesses were determined with X‐ray reflectivity. Finally, in order to prevent them from oxidation, all films were capped in situ with a 10 nm‐thick either amorphous SiN_x_ or polycrystalline TiN layer.

### 5.2 Laboratory X‐ray Diffraction Measurements

Diffraction patterns were acquired in the *θ*–2*θ* geometry using a D8 Bruker diffractometer equipped with a Ge monochromator to select the Cu (K_*α*1_) radiation (*λ* = 1.5406 Å) and in‐plane geometry, using a Rigaku SmartLab diffractometer (Cu K_*α*1_ and K_*α*2_ radiations). Rocking curves (*ω* scans at a fixed 2*θ* value) were also measured on selected Bragg diffraction peaks.

### 5.3 Anomalous X‐ray Diffraction measurements

#### 5.3.1 Experiment

Anomalous X‐ray diffraction measurements in the energy range [11–11.25 keV] around the Ge K‐edge were performed at Synchrotron SOLEIL on the DiffAbs beamline, using a six‐circle diffractometer configured in kappa geometry [48]. The X‐ray beam was monochromatized using a Si(111) monochromator (*ΔE/E* ≈ 10^−4^). Harmonics rejection (≈10^−6^) was ensured by two Rh‐coated flat mirrors in grazing geometry. The vertical size (FWHM) of the incident X‐ray beam in the scattering plane was 0.16 mm and its horizontal size 0.26 mm. A two‐dimensional (2D) XPAD hybrid pixel detector was used to record the scattered intensity [49, 50]. 2D images of the 0 0 *l* Bragg diffraction peaks (*l* = 3, 6, 9, 12) for the 100 nm‐thick seeded GeTe film deposited on SiO_2_ were acquired for X‐ray energy varying in steps of 1 eV. The intensity of the incident beam was measured simultaneously. The distance between the center of the goniometer and the 2D detector was chosen so that the ranges of the scattering angle 2*Ɵ* (±5.5°) and azimuth angle *Ψ* (±7.5°) of the 2D detector exceed the extension of the diffraction peak (see Figure S8a). For a given *l* value, the angular position of the 2D detector could be kept constant as a function of the energy but the angle between the incident beam and the film surface was changed so that at each energy *ω* = 2*θ_c_* /2 with 2*θ_c_* the scattering angle at the peak maximum. This is mandatory to obtain a reliable measurement of the scattered intensity in a *c*‐oriented film. The *ω* values have been calculated prior to the measurement from the known lattice parameter. In the 2*θ* range where the scattered intensity is non‐zero, the *ω*‐offset (2*θ* /2*‐ω*) remains small compared to the width of the rocking curve (see Figure S8c,d). The raw images acquired with the 2D detector were analyzed in order to obtain the integrated intensity of each Bragg peak as a function of the energy as explained in Supporting Information S1: Section 4.1. The sensitivity of the 2D detector can be considered as constant in the energy range measured. The integrated intensity was then divided by the energy‐dependent incident beam intensity.

#### 5.3.2 Simulations

The integrated intensity of a diffraction peak is proportional to the square modulus of the unit cell structure factor *F*_00*l*_(*E*). For 0 0 *L* peaks in *α*‐GeTe, using the hexagonal unit cell with Te atoms in (0, 0, 0), (1/3, 2/3, 2/3) and (2/3, 1/3, 1/3) and Ge atoms in (0, 0, 1/2+δ), (1/3, 2/3, 1/6+δ), and (2/3,1/3,5/6+ δ) relative positions (see Supporting Information S1: Section 1) the unit cell structure fact is:

(1) $$F_{00l}\left(E\right)=3f_{Te}\left(l,E\right)e^{-M_{Te}}+3f_{Ge}\left(l,E\right)e^{-M_{Ge}}e^{i2\pi l\left(0.5+\delta \right)}$$

where $e^{-M_{Ge}}$ and $e^{-M_{Te}}$ are Debye‐Waller factors taking into account the effect of atomic vibrations, *f_Ge_* (*l*,*E*) and  *f_Te_* (*l*,*E*) are the atomic scattering factors for Ge and Te. They are the sum of the Thomson atomic scattering factor *f* ^0^, which depends on the norm of the scattering vector $Q=\frac{2\;\pi \;l}{c_{h}}$ but is independent of *E*, and dispersion (anomalous) terms *f*′(*E*) + *i* *f*′′(*E*) which can be considered as independent of the scattering vector [35, 36]. *f*′′(*E*) is proportional to the photoelectric absorption coefficient and increases abruptly as *E* increases above an absorption edge energy. For energy above the edge *f’*(*E*) and *f”*(*E*) are the sum of a term varying smoothly with E (bare atom contribution) and an oscillating contribution, existing in an energy range of about 1 keV above the edge energy. The latter depends on the local environment of the absorbing atom. It is not considered in the present work. Values of dispersion terms for isolated atoms Ge and Te atoms [51] were thus used for calculations of |*F*_00*l*_|^2^. Values of the Thomson atomic scattering factors were taken from reference [52].

The integrated intensity of a 0 0 *l* diffraction peak *I*_00*l*_(*E*) divided by the incident beam intensity *I_inc_* (*E*) is proportional to |*F*_00*l*_(*E*)|^2^.The proportionality factor is *KP*(θ)*L*(θ)*A*(*E*, θ) where *K* is an overall scaling factor, *L*(θ) a geometrical correction (Lorentz factor), *A*(*E*, θ) describes absorption corrections [34, 38, 53]. The polarization correction *P*(θ) is equal to 1 because, in the geometry used, the polarization of the incident beam is perpendicular to the scattering plane. Absorption corrections are minor for the film studied, of the order of a few percent. For comparison with experimental results in Figure 4 the calculated |*F*_00*l*_(*E*)|^2^ values were multiplied by *L*(θ)*A*(*E*, θ) and then normalized.

### 5.4 Scanning Transmission Electron Microscopy

Lamellae for transmission electron microscopy (TEM) analysis were prepared using a Ga^+^ focused ion beam (FIB) with a ThermoFisher Scientific Helios 450 system. Initial milling was performed at 16 kV with medium beam current, followed by low‐energy polishing (5–8 kV) to minimize ion‐induced damage, resulting in final lamella thicknesses of approximately 60 nm. High‐angle annular dark‐field (HAADF) scanning transmission electron microscopy (STEM) imaging was carried out on a probe Cs corrected FEI Titan Themis microscope operating at 200 kV. To reduce electron beam‐induced damage, HAADF images were acquired using a low current (<30 pA). Precession electron diffraction (PED), also referred to as automated crystal orientation mapping (ACOM), was employed to analyze the micro‐texture of the films. PED measurements were conducted on a JEOL NeoARM200F microscope at 200 kV, equipped with a NanoMEGAS ASTAR system. The microscope was operated in nanobeam diffraction large (NBD‐L) mode with a 1 nm probe size and a 10 µm condenser aperture. Diffraction patterns (512 × 512 pixels) were recorded using a Stingray CCD camera, with the camera length calibrated against the silicon substrate. Each pattern was acquired with a 30 ms integration time.

### 5.5 Piezoresponse Force Microscopy

Vertical Piezoresponse Force Microscopy (PFM) measurements were carried out at room temperature using a Bruker Dimension Icon AFM installed in a glove box maintained under argon atmosphere (O_2_ and H_2_O concentrations in the ppm range). The piezoresponse induced by the applied tip‐sample AC voltage is measured through the tip height variations, with the sample being electrically grounded. The experiments were performed in PFM Dual Frequency Resonance Tracking (PFM‐DFRT) mode in order to track the tip contact resonance frequency to increase the measured piezoresponse. Prior to these measurements, a ±10 V DC voltage was applied locally to pole square regions. All regions were written at a same tip scanning speed. Switching Spectroscopy PFM‐DFRT (SSPFM‐DFRT) mode was used to probe the ferroelectric behavior [54]. In this mode, a DC voltage of variable amplitude is cyclically applied to the tip. The piezoresponse is recorded for each cycle at zero DC voltage with the DFRT mode. An AC excitation of 2 V and 10 ms voltage pulses between −10 and +10 V is applied. We used platinum coated either silicon or platinum‐iridium tips (SCM‐PtSi and SCM PIT‐V2 from Bruker) with spring constant around 3 N.m^−1^ and resonance frequency in the ranges of 50‐100 kHz and 75‐100 kHz, respectively.

## Funding

This work was supported in part by the PEPR Electronique through the France 2030 government grants EMCOM (ANR22‐PEEL‐0009).

## Conflicts of Interest

The authors declare no conflicts of interest.

## Supporting information




**Supporting File**: advs75711‐sup‐0001‐SuppMat.docx.

## Data Availability

The data that support the findings of this study are available from the corresponding author upon reasonable request.
