# Supplementary material for: Large‐Scale Growth of Self‐Poled Ferroelectric Rashba Semiconductor α‐GeTe(111) Thin Films: A Crucial Step Towards Future CMOS‐Compatible Ferroelectric Spintronic Devices
Source: Adv Sci (Weinh). 2026 May 22;13(47):e75711. doi: 10.1002/advs.75711 (PMC13336094; doi:10.1002/advs.75711)
Supplement: Supplementary file 1 [file ADVS-13-e75711-s001.docx]

Supporting Information

Large-Scale Growth of Self-Poled Ferroelectric Rashba Semiconductor *α*-GeTe(111) Thin Films: A Crucial Step Towards Future CMOS-Compatible Ferroelectric Spintronic Devices

Jules Lagrave, Nicolas Bernier, Thomas Jalabert, Yoann Brûlé, Damien Térébénec, Pierre Meilleur, Hervé Roussel, Jean-Baptiste Dory, Oussama J. Mouawad, Thibault Chommaux, Cristian Mocuta, Dominique Thiaudière, Laurent Vila, Francoise Hippert and Pierre Noé^*^

**^*^Contact:** [pierre.noe@cea.fr](mailto:pierre.noe@cea.fr)

1. *α*-GeTe structure description

The rhombohedral *α*-GeTe phase (space group R3m) can be described as a distorted NaCl-type cubic structure, elongated along one the <1 1 1> cubic directions. This distortion arises from the relative displacement of the Ge and Te sublattices along a cubic diagonal, which breaks inversion symmetry and gives rise to a ferroelectric polarization aligned with the elongated diagonal, oriented in one direction or the other. Hence, there are eight possible polarization directions. The *α*-GeTe structure can be equivalently described using three crystallographic unit cells (see **Figure S1a**): a primitive rhombohedral cell containing one Ge and one Te atom (lattice parameters *a_r_*, *α_r_*), a distorted *pseudo*-cubic cell containing four Ge and four Te atoms (lattice parameters *a*_c_, *α*_c_) or a hexagonal unit cell containing three Ge and three Te atoms (lattice parameters *a_h_*, *c_h_*). The *c*-axis of the hexagonal unit cell coincides with the elongated *pseudo*-cubic diagonal. The link between the lattice parameters of the *pseudo*-cubic and hexagonal cells is given thereafter.

$$\begin{aligned} a_{c}=\sqrt{\frac{4}{3}a_{h}^{2}+\frac{1}{9}c_{h}^{2}}\#\left( 1 \right) \end{aligned}$$

$$\begin{aligned} \cos{(\alpha}_{c})=\frac{c_{h}^{2}-6a_{h}^{2}}{c_{h}^{2}+12a_{h}^{2}}\#\left( 2 \right) \end{aligned}$$

$$\begin{aligned} c_{h}=a_{c}\sqrt{3+6\cos\left( \alpha_{c} \right)}\#\left( 3 \right) \end{aligned}$$

$$\begin{aligned} a_{h}=a_{c} \sin\left( \alpha_{c}/2 \right)\#\left( 4 \right) \end{aligned}$$

In the case of a *α*-GeTe(111) film, the hexagonal unit cell (**Figure S1b**) provides an intuitive description of the structure because its c-axis is perpendicular to the film and the ferroelectric polarization is aligned with the *c*-axis. Te atoms occupy the (0, 0, 0), (1/3, 2/3, 2/3), and (2/3, 1/3, 1/3) atomic positions and Ge atoms the (0, 0, 1/2+$\delta$), (1/3, 2/3, 1/6+$\delta$), and (2/3, 1/3, 5/6+$\delta$) positions. $\delta$ decreases as the temperature increases and reaches zero at the rhombohedral-cubic transition ^[1]^. Two consecutive Te planes perpendicular to the *c*-axis are at a distance of *c_h_*/3. The Ge atoms are shifted vertically along the *c*-axis by *d_Ge_* = $\delta$*×c_h_*, which leads to distances between the Ge and Te planes equal to (1/6+$\delta$) *c_h_* and (1/6-$\delta$) *c_h_*. The polarization is pointing upward if the Ge displacement *δ* is positive and downward if *δ* is negative (**Figure S1c** and **S1d)**.

**Figure S1.** a) Illustrations of the *α*-GeTe structure in the *pseudo*-cubic (green lines), rhombohedral (red lines) and hexagonal (black lines) unit cells. b) *α*-GeTe hexagonal unit cell represented alone for easier visualization with *a_h_* = 4.1639 Å, *c_h_* = 10.6922 Å and $\delta$ = 0.025, which are the values of Ref. [1] for a GeTe single crystal at 295 K. The Ge displacement *d_Ge_* being positive, the ferroelectric polarization, aligned with the *c*-axis, is pointing upward. Projections of the structure along the zone axis <2 1 0>, which corresponds to the zone axis of the HAADF-STEM image in **Figure 3c** of the main text, for $\delta$ = 0.025 c) and $\delta$ = -0.025 d). In b), c) and d) the ferroelectric polarization direction is indicated with a red arrow.

2. Laboratory X-ray diffraction

2.a. In-plane diffraction:

In-plane X-ray diffraction patterns were acquired at an incident angle of 0.35° using Cu K_α1_ and K_α2_ radiations in the 2*θχ* range [20-100°] for the three seeded GeTe films whose out-of-plane diffraction patterns are shown in **Figure 1a** of the main text. The detected diffraction peaks can all be indexed as *h k* 0 reflections (hexagonal indexation) of *α*-GeTe, consistent with the out-of-plane diffraction data and confirming that the *c*-axis is perpendicular to the substrate. Diffraction peaks from the Sb_2_Te_3_ seed layer are not observed, as the probed thickness at this incident angle is limited to ≈ 20 nm. The thickness of the films deposited on a-Si and SiO_2_ is 100 nm and that of the film deposited on TiN 25 nm. In the latter case, the film was capped with a TiN layer, leading to the appearance of 1 1 1 and 2 0 0 TiN diffraction peaks in the diffraction pattern. The intensity of the GeTe 1 1 0 reflection was measured during a *Φ*-scan (rotation around the normal to the film at 2*θχ* constant). One example is shown in **Figure S2b**. The measured intensity does not depend on *Φ*, showing that the crystallites have random in-plane orientations and the three studied films have a fiber texture.

**Figure S2.** a) In-plane diffraction pattern (Cu K_α1_ and K_α2_ radiations), acquired for an incident angle of 0.35°, on 100 nm thick Sb_2_Te_3_ seeded GeTe films deposited on SiO_2_ and a-Si and a 25 nm thick Sb_2_Te_3_ seeded GeTe films deposited on TiN. The curves have been shifted vertically for clarity. Only *h* *k* *0* diffraction peaks (hexagonal indexation) of the *α*-GeTe phase are detected. The probed thickness is about 20 nm. The 1 1 1 and 2 0 0 TiN diffraction peaks observed for the seeded GeTe film deposited on TiN are due to the presence of a 10 nm thick TiN capping layer. The two other films are capped with a 10 nm amorphous SiN_x_ layer. b) *Φ* scan for the GeTe 1 1 0 peak (2*θχ*=43.25°) of the seeded GeTe film deposited on SiO_2_. The incident angle is 1.4° in order to suppress the influence of the rectangular shape of the sample.

2.b. Rocking curves:

Rocking curves of the GeTe 0 0 6 and Sb_2_Te_3_ 0 0 15 reflections were measured for the seeded GeTe film grown on SiO_2_ (see **Figure S3a** and **S3b**). To compare them, each rocking curve, measured at a fixed 2𝜃 value, has been normalized (maximum intensity set to 1) and drawn a as a function of *ω - ω_c_*, with *ω_c_* the value of *ω* for the maximum of the curve (see **Figure S3c**). Notably, the FWHM of the GeTe 0 0 6 reflection closely follows that of the Sb_2_Te_3_ 0 0 15 reflection, indicating that the orientation of the GeTe crystallites is determined by that of the Sb_2_Te_3_ crystallites in the seed layer. The same conclusion applies to the seeded GeTe film deposited on TiN (**Figure S3d**). In the latter case, the rocking curves are larger than for the film deposited on SiO_2_**.**

**Figure S3.** Rocking curves (*ω* scans) of the a) Sb_2_Te_3_ 0 0 15 and b) GeTe 0 0 6 reflections for the seeded GeTe film grown on SiO_2_. Comparison of the normalized rocking curves of the GeTe 0 0 6 and Sb_2_Te_3_ 0 0 15 reflections for the seeded-GeTe films grown c) on SiO_2_ and d) on TiN. Each rocking curve, measured at a fixed 2𝜃 value, has been normalized (maximum intensity set to 1) and drawn as a function of *ω - ω_c_*, with *ω_c_* the value of *ω* for the maximum of the curve.

2.c Comparison of GeTe films deposited on SiO_2_, with and without a Sb_2_Te_3_ seed layer:

Out-of-plane X-ray diffraction patterns (*λ* = 1.5406 Å, Cu K_α1_ radiation) were recorded in *θ*–2*θ* geometry for 100 nm thick GeTe films deposited on SiO_2_, with and without a Sb_2_Te_3_ seed layer (**Figure S4a**). In both cases, the diffraction peaks are indexed as 0 0 *l* reflections, indicating a GeTe(111) oriented growth with a ferroelectric polarization perpendicular to the film surface (*c*-oriented). However, the rocking curve of the GeTe 0 0 6 reflection shows a marked difference between seeded and unseeded films. The FWHM is 2.5 times larger in the unseeded film (**Figure S4b**). In the case of the unseeded film, a significant intensity is measured on both sides of the rocking curve. This is not a background signal, as demonstrated by out-of-plane diffraction patterns acquired with an *ω* offset of 10° (**Figure S4c** and **S4d**). A diffraction peak with a significant intensity is measured in the unseeded film, not in the seeded one. This confirms the presence of an appreciable amount of misoriented crystallites in the unseeded sample.

**Figure S4.** a) Out-of-plane diffraction patterns (λ = 1.5406 Å, Cu K_α1_ radiation), acquired in the *θ*/2*θ* geometry of seeded and unseeded 100 nm thick GeTe films deposited on SiO_2_. The GeTe and Sb_2_Te_3_ peaks are indexed as 0 0 *l* peaks (hexagonal indexation of the rhombohedral phases). b) Rocking curves of the GeTe 0 0 6 reflection for the seeded and unseeded films. The corresponding 2𝜃 angles are 51.385° and 51.222° respectively. Comparison of out-of-plane diffraction patterns acquired the *θ*/2*θ* geometry with patterns acquired with an *ω*-offset of 10° in the 2𝜃 range corresponding to the 0 0 6 reflection for the c) seeded and d) unseeded films. The *ω*-offset is indicated by colored vertical lines in b).

2.d Statistical analysis at the wafer scale:

**Figure S5.** a) Characteristic out-of-plane X-ray diffraction patterns and b) Rocking curves on 0 0 6 diffraction peak of a 100 nm thick GeTe film seeded with Sb_2_Te_3_ deposited on a-Si acquired along the radius of a 300 mm diameter wafer. The slight increase of the FWHM of the rocking curve along the wafer radius is correlated to a slight increase of the rocking of Sb_2_Te_3_ seed layer. It most probably results from a small temperature gradient (estimated to ~10-20°C) in our 300 mm sputtering deposition tool.

**Figure S6.** WDXRF mapping of relative composition change of a 100 nm thick GeTe film seeded with Sb₂Te₃ deposited on thermal SiO_2_ over a 300 mm diameter wafer. The reference GeTe composition was measured at the center of the wafer, and the maps were produced by plotting the deviation in atomic % of a) Ge and b) Te compositions from the reference values, measured at 2 cm intervals along the radius of the wafer.

3. Interface between the Sb_2_Te_3_ seed layer and GeTe

A HAADF-STEM image of the interface between the Sb_2_Te_3_ seed layer and GeTe is shown in **Figure S7a**. It was acquired in the same run as the images shown in **Figure 3d** of the main text. 4 Sb_2_Te_3_ QLs separated by *pseudo* vdw gaps are clearly visible in the bottom of the image while Ge and Te planes are detected in the upper part of the image. The gap located above the fourth QL is associated with a 60° in-plane rotation (twin) of the upper atomic plane. No further gaps are detected in the upper portion of the image. In order to examine in details the transition between the Sb_2_Te_3_ seed layer and the GeTe layer, linear intensity profiles were calculated for atomic columns located along the three arrows drawn in **Figure S7a**. They are shown in **Figure S7b**. Due to the contrast formation of the HAADF image, the intensity of the different peaks in the linear profile increases with Z of the atoms of the corresponding column. The black profile characterizes the Sb_2_Te_3_ layer. It shows close intensity peaks, corresponding to Sb and Te atomic columns, and *pseudo* vdw gaps between Te peaks. The higher intensity of the central Te column, inside the Sb_2_Te_3_ QL, compared to that of the outer Te columns, close to gaps, is in line with previous observations. It can be explained by reduced vibrational motion of the central Te atoms compared to the outer ones.^[2]^ The blue profile shows a succession of low and high intensity peaks. The intensity of the weak peaks is constant along the profile. Consistent with the analysis of the HAADF-STEM image, the weak intensity peaks correspond to Ge columns. Thus, the blue profile characterizes the GeTe layer. The red profile therefore allows to visualize the transition between the Sb_2_Te_3_ and GeTe layers. Starting from the left, a progressive change from high intensity peaks, corresponding to pure Sb columns (like in the black profile), to weak intensity peaks, corresponding to pure Ge columns (like in the blue profile), is observed. This clearly indicates a Sb/Ge intermixing which extends on about 2-3 nm in the direction normal to the planes. The transition between a pure Sb_2_Te_3_ layer and the GeTe layer is therefore gradual. The deposition time was chosen to deposit 5 Sb_2_Te_3_ QLs. The present results show that Ge atoms have diffused during deposition into the last deposited Sb_2_Te_3_ QL and that the subsequent growth of GeTe occurs without the formation of a Te-Te gap.

**Figure S7.** a) HAADF-STEM image of a Sb_2_Te_3_ seeded GeTe film grown on SiO_2_. Linear intensity profiles were calculated for atomic columns located along three arrows (black arrow in the Sb_2_Te_3_ layer, blue arrow in the GeTe layer and red arrow in the transition zone). b) Obtained profiles vertically shifted for clarity.

4. Anomalous X-ray diffraction at the Ge K-edge using synchrotron radiation

4.1 Data treatment:

The raw 2D images acquired with the XPAD detector were corrected following the procedure described in Ref. [3] in order to obtain the scattered intensity as a function of the scattering angle 2*θ* and the azimuthal angle *ψ, i.e.* the azimuthal position on the diffraction ring taking the vertical scattering plane as *ψ=0°*. An example of the obtained 2D intensity map is given in **Figure S8a** for the 0 0 6 reflection for the seeded GeTe film deposited on SiO_2_. The scattered intensity has been divided by the incident beam intensity. Integration of the 2D map in a 2*θ* range of 0.1° centered around the scattering angle of the peak maximum leads to the curve shown in **Figure S8b**. Its FWHM is equal to 2.17(2)°. This is almost identical to the FWHM of the rocking curve (*ω* scan) of the 0 0 6 reflection in the same film (**Figure S3b**), as expected in the presence of a fiber texture.

The integrated intensity of a diffraction peak is obtained in two steps. First, at each $2\theta$ value, an integration on the whole *ψ* range of the 2D map is performed. Examples of the obtained curves for the 0 0 6 and 0 0 9 reflections are given in **Figures S8c** and **S8d** for two X-ray energies, smaller and larger than the Ge K-edge energy *E_0_* equal to 11.1026 keV in the film studied. All curves can be satisfactorily adjusted to a Pearson VII function including a linear baseline. This baseline is subtracted and the area under the resulting curve is calculated to obtain the integrated intensity of the diffraction peak. Subtracting the background eliminates the contribution of fluorescence radiation for energies above the K-edge energy.

**Figure S8.** a) 2D map of the scattered intensity as a function of 2*θ* and *ψ*, deduced from the 2D image acquired with the XPAD detector, for the 0 0 6 reflection at a photon energy *E* =11.0 keV for the seeded GeTe film deposited on SiO_2._ The acquisition time is equal to 1 s. The measured intensity has been divided by the incident beam intensity *I_Inc_*. The units are arbitrary, the scattered intensity and the incident one being measured by different detectors. b) *ψ* dependence of the intensity obtained by integration of the 2D map in a) in a $2\theta$ range of 0.1° centred at $2\theta_{\max}$ = 37.035°. c) $2\theta$ dependence of the intensity, after integration on the whole *ψ*range of the 2D map for each 2*θ* value, for the 0 0 6 reflection at *E* = 11.0 keV below the Ge K-edge energy and 11.25 keV above the edge energy. The $2\theta$ step is 0.01914°. At each energy, the angle of the incident beam with the film surface $\omega$ was set so that $\omega=2\theta_{\max}/2$ with $2\theta_{\max}$ the scattering angle at the peak maximum. This ensures that the scattering geometry is close to a symmetric $\theta/2\theta$ configuration for the whole peak. For example, for E= 11 keV at $2\theta$*=* 37.6°, on the right of the peak, the *ω*-offset ($2\theta$/2*-*$\omega$) is equal to 0.25°, which is small compared to the width of the rocking curve shown in **Figure S3b**. d) Same as c) for the 0 0 9 reflection. Increase in the background intensity for energy above the edge is due to fluorescence radiation.

4.2 Calculation of the unit cell structure factor including dispersion terms:

For the hexagonal unit cell of *α*-GeTe,

$$\begin{aligned} F_{00l}\left( E \right)={3 f}_{Te}\left( l,E \right)e^{-M_{Te}}+3 f_{Ge} \left( l,E \right)e^{-M_{Ge}} e^{i2\pi l\left( 0.5+\delta\right)},\#\left( 5 \right) \end{aligned}$$

where the factors $e^{-M_{Ge}}$ and $e^{-M_{Te}}$ are the Debye-waller factors. $M_{i}=\frac{1}{2} Q^{2}<{u_{i\boldsymbol{Q}}}^{2}>$, with $<{u_{i\boldsymbol{Q}}}^{2}>$ the mean-square projection of the atomic displacement of atom *i* on the direction of the scattering vector *Q.*^[4,5]^ For isotropic atomic vibrations, as commonly assumed for Ge and Te atoms in GeTe crystals,^[1,6]^, $u_{\boldsymbol{Q}}$ does not depend on the direction of *Q*. For a given atom, the atomic scattering factor *f* is the sum of $f^{0}$ the Thomson atomic scattering factor, which depends on the norm of the scattering vector $Q=\frac{2 \pi l}{c_{h}}$ but is independent of X-ray energy *E,* and dispersion (anomalous) terms *f’*(*E*)+ i *f”*(*E*) which depend on E but are independent of the scattering vector.^[5,7]^

$$f_{Ge}\left( l,E \right)=f_{Ge}^{0}\left( l \right)+f_{Ge}^{'}(E)+if_{Ge}^{''}(E) (6)$$

$f_{Te}\left( l,E \right)=f_{Te}^{0}\left( l \right)+f_{Te}^{'}(E)+if_{Te}^{''}(E)$	(7)

Values of the Thomson atomic scattering factors of Ge and Te $f_{Ge}^{0}\left( l \right)$ and $f_{Te}^{0}\left( l \right)$ were taken from reference [8]. The dispersion terms for isolated atoms Ge and Te atoms were taken from reference [9].

When dispersion terms can be neglected, $\left| F_{00l} \right|^{2}$ does not depend on *E*.

$$\begin{aligned} \left| F_{00l} \right|^{2}=\left[ {3 f}_{Te}^{0}(l)e^{-M_{Te}}+{3 \left( -1 \right)^{l}f}_{Ge}^{0}(l)e^{-M_{Ge}} \cos\left( 2\pi l\delta\right) \right]^{2}+\left[ {3 f}_{Ge}^{0}(l)e^{-M_{Ge}}\sin\left( 2\pi l\delta\right) \right]^{2}.\#\left( 8 \right) \end{aligned}$$

$\left| F_{00l} \right|^{2}$ does not depend on the sign of $\delta$.

For energies close to the that of the Ge K-edge, dispersion terms must be included and $\left| F_{00l} \right|^{2}$ depends on *E* and on the sign of $\delta$.

$\left| F_{00l up}(E) \right|^{2}-\left| F_{00l down}\left( E \right) \right|^{2}=$

$$-36\times\left( -1 \right)^{l}\times\left[ {{(f}_{Te}^{0}\left( l \right)+f}_{Te}^{'}(E))f_{Ge}^{''}(E)-f_{Te}^{''}{(E) (f}_{Ge}^{0}(l)+f_{Ge}^{'}(E)) \right] e^{-M_{Ge}}e^{-M_{Te}}( \sin\left( 2\pi l\left| \delta\right| \right) (9)$$

with $\left| F_{00l up}(E) \right|^{2}$ the square modulus of the structure factor for a uniformly upward polarized state ($\delta>0$) and $\left| F_{00l down}\left( E \right) \right|^{2}$ the square modulus of the structure factor for a uniformly downward polarized state ($\delta<0$). For *E* larger than the Ge K-edge energy, $\left| F_{00l up}(E) \right|^{2}<\left| F_{00l down}\left( E \right) \right|^{2}$ if *l* is even $\left| F_{00l up}(E) \right|^{2}>\left| F_{00l down}\left( E \right) \right|^{2}$ if if *l* is odd, as can be seen in **Figure S9**.

**Figure S9.** Integrated intensity for 0 0 *9* diffraction peak, divided by the incident beam intensity $I_{inc}$ and normalized to 1 for *E* =11 keV (black curve), for the 100 nm thick seeded GeTe film deposited on SiO_2_. Calculated intensities, normalized to 1 for *E* =11 keV, are drawn assuming a film entirely polarized upwards (curves colored with warm colors), entirely polarized downwards (curves colored with cold colors) and a film without macroscopic polarization containing equal volumes of ferroelectric domains oriented in one direction or the other (grey curve). The peak observed for $E\approx E_{0}$is due to the near-edge fine structure. Calculations were performed for different positive and negative$\delta$ values. The Ge relative coordinate in the hexagonal unit cell is $0.5+\delta$. A positive $\delta$ value corresponds to a polarization pointing upward and a negative one to a polarization pointing downward. The best agreement between experimental and calculated intensities is obtained for $\delta$= 0.025.

**Figure** **S10.** Integrated intensities for 0 0 6 and 0 0 9 diffraction peaks, divided by the incident beam intensity $I_{inc}$ and normalized to 1 for *E* =11 keV (black curves), for a 100 nm thick GeTe film deposited on SiO_2_ without any seed layer. Calculated intensities, normalized to 1 for *E* =11 keV, are drawn assuming a film entirely polarized upward (*P_Up_*, red curves), entirely polarized downward (*P_Down_*, blue curves) and a film without macroscopic polarization containing equal volumes of ferroelectric domains oriented in one direction or the other (50%, grey curves). The peak observed for $E\approx E_{0}$is due to the near-edge fine structure. Calculations were performed for $\delta$=+0.025 or -0.025. The Ge relative coordinate in the hexagonal unit cell is $0.5+\delta$ . A positive $\delta$ value corresponds to a polarization pointing upward and a negative one to a polarization pointing downward. The noise in the experimental signal is attributed to the limited number of *c*-axis oriented crystallites present in this unseeded sample.

5. Piezoresponse Force Microscopy (PFM):

Vertical PFM was performed on a 40 nm thick Sb_2_Te_3_ film grown on an a-Si substrate and capped with a 10 nm thick SiN_x_ layer. Amplitude and phase hysteresis loops were recorded using SSPFM-DFRT mode (**Figure S11**). No piezoelectric response was detected in the SSPFM measurements.

**Figure S11.** Amplitude and phase hysteresis loops obtained in SSPFM-DFRT mode for a 40 nm thick Sb_2_Te_3_ film grown on an a-Si substrate and capped with a 10 nm thick SiN_x_ layer.

**References**

[1]	T. Chattopadhyay, J. X. Boucherle, H. G. von Schnering, *J. Phys. C: Solid State Phys.* **1987**, *20*, 1431.

[2]	V. Sever, N. Bernier, D. Térébénec, C. Sabbione, J. Paterson, F. Castioni, P. Quéméré, A. Jannaud, J.-L. Rouvière, H. Roussel, J.-Y. Raty, F. Hippert, P. Noé, *physica status solidi (RRL) – Rapid Research Letters* **2024**, *18*, 2300402.

[3]	C. Mocuta, M.-I. Richard, J. Fouet, S. Stanescu, A. Barbier, C. Guichet, O. Thomas, S. Hustache, A. V. Zozulya, D. Thiaudière, *Journal of Applied Crystallography* **2013**, *46*, 1842.

[4]	K. N. Trueblood, H. B. Bürgi, H. Burzlaff, J. D. Dunitz, C. M. Gramaccioli, H. H. Schulz, U. Shmueli, S. C. Abrahams, *Acta Crystallogr A Found Crystallogr* **1996**, *52*, 770.

[5] Jens Als-Nielsen, Des McMorrow, In *Elements of Modern X-Ray Physics*, John Wiley & Sons, Ltd, **2011.**

[6] M. Sist, H. Kasai, E. M. J. Hedegaard, B. B. Iversen, *Phys. Rev. B* **2018**, *97*, 094116.

[7] H. Renevier, M. G. Proietti, in *International Tables for Crystallography Vol. I*, edited by C.T. Chantler, F. Boscherini and B. Bunker, John Wiley & Sons, Ltd, **2024**, pp. 201-204

[8]	P. J. Brown, A. G. Fox, E. N. Maslen, M. A. O’Keefe, B. T. M. Willis, in *International Tables for Crystallography Vol.C*, edited by E. Prince, John Wiley & Sons, Ltd, **2006**, pp. 554–595.

[9]	“CXRO X-Ray Interactions With Matter,” can be found under https://henke.lbl.gov/optical_constants/
